# Critical factors influencing the adoption of virtual reality technology for improving mental health among university students: an extended technology acceptance model

**DOI:** 10.3389/fpsyg.2026.1799274

**Published:** 2026-03-18

**Authors:** Da Xing, Yubo Zhou, Xianghui Wei, Zhen Qin

**Affiliations:** 1 School of Information Technology and Engineering, Guangzhou College of Commerce, Guangzhou, China; 2 School of Computer Science, Beijing Institute of Technology, Beijing, China; 3 School of Design, South China University of Technology, Guangzhou, China

**Keywords:** mental health, structural equation modeling, technology acceptance, university students, virtual reality

## Abstract

Virtual reality (VR) technology has garnered substantial research interest as a tool for university student mental healthcare. To understand the drivers of its adoption among university students, this study aimed to introduce an extended technology acceptance model incorporating facilitating conditions, social influence, and perceived enjoyment. Based on survey data from 327 university students, analyzed via structural equation modeling, the study validated the model’s applicability in this context. Results revealed that while facilitating conditions and perceived enjoyment significantly enhance perceived usefulness and perceived ease of use, social influence specifically impacts perceived usefulness. 74.0% of the variance in behavioral intention to use VR technology to improve mental health among university students is explained by the model. The findings provided a good understanding of VR technology acceptance among university students for improving mental health. Based on the findings, practical recommendations are discussed to optimize the deployment of VR-based mental health interventions for university students.

## 1 Introduction

The mental well-being of university students has become a significant challenge for higher education institutions globally (Ozamiz-Etxebarria et al., 2025; Shi et al., 2025). Approximately 84% of university students experienced stress (Abulfaraj et al., 2024), 31% of university students tested positive for at least one mental disorder within the past 12 months (Auerbach et al., 2018), and 25% of university students have experienced suicidal ideation (Mortier et al., 2018). University students navigate a high-pressure environment characterized by academic rigor, financial stress, and complex social–emotional transitions, all of which are significant contributors to psychological distress (Mu et al., 2022). While traditional counseling services are essential, university students face persistent challenges of scalability, stigma, and accessibility, underscoring the critical need for innovative, engaging, and effective interventions (Cerolini et al., 2023). In this context, virtual reality (VR) technology has garnered substantial research interest as a tool for university student mental healthcare (Man et al., 2025d).

VR technology can provide a computer-generated, interactive, and three-dimensional environment experienced through a head-mounted display, isolating users from external stimuli and immersing them in a virtual setting (Man et al., 2024). The therapeutic potential of VR technology is predicated on its unique psychological characteristics. The primary of these characteristics is the induction of presence, which is a compelling subjective sensation of being there in the virtual environment. This sense of presence allows VR technology to serve as a powerful tool for interventions that bridge the gap between artificial clinical settings and unpredictable real-world scenarios (Bell et al., 2024). The key characteristics of VR technology make it exceptionally well-suited for mental health applications. Its capacity for controlled and graded exposure allows for the safe simulation of anxiogenic stimuli, forming the basis of VR exposure therapy in the treatment of phobias and anxiety disorders (Kuleli et al., 2025). Furthermore, VR technology enables the creation of immersive and calming environments for mindfulness and stress-reduction exercises (Olasz et al., 2024) and the simulation of complex social interactions for skills training (Li et al., 2025). By offering a private, interactive, and customizable experience, VR technology has the potential to overcome many barriers that deter students from seeking traditional forms of help.

Although VR technology offers advantages for university students’ mental health, its mere availability does not guarantee effective adoption. Previous studies have investigated students’ technology acceptance to learn English (Man et al., 2025b). However, no studies have examined university students’ acceptance of VR technology to improve mental health. Therefore, this study aimed to identify the determinants affecting university students’ acceptance of VR technology to improve mental health. Specifically, this study proposed a research model integrating the technology acceptance model (TAM), facilitating condition (FC), social influence (SI), and perceived enjoyment (PE). The findings were expected to enhance understanding of how FC, SI, and PE influence students’ acceptance of VR technology to improve mental health. Moreover, this study would assist VR technology developers, educators, and policymakers in designing effective interventions to increase students’ acceptance of VR technology for improving mental health.

## 2 Literature review

### 2.1 TAM

Originally proposed by Davis (1989), the TAM remains a cornerstone framework for explaining information system adoption. Central to this model is the premise that individuals’ behavioral intention to use (BITU) is driven by perceived usefulness (PU) and perceived ease of use (PEOU), both of which, alongside their direct interrelationships, influence attitude toward using (ATU). Adapted to the present context, PU reflects the belief that VR technology facilitates mental health improvement, PEOU denotes the expectation of an effort-free experience. ATU and BITU represent the respective affective stance and intentional intensity toward this technology. Given the TAM’s extensive validation in educational settings, ranging from e-learning platforms (Sharma et al., 2024) to mobile applications (Liu et al., 2024). Thus, the hypotheses were developed:

*H1*: PEOU positively affects ATU.

*H2*: PEOU positively affects PU.

*H3*: PU positively affects ATU.

*H4*: PU positively affects BITU.

*H5*: ATU positively affects BITU.

### 2.2 FC

FC is formally defined as the degree to which individuals believe that organizational and technical infrastructures exist to help their use of a technology (Man et al., 2022). FC reflects the availability of technical and organizational resources supporting technology use. Previous technology acceptance studies showed that FC positively influences PU and PEOU in the context of highly automated vehicles for the public (Man et al., 2025c), cloud-based academic information systems for students (Wandira et al., 2024), and ChatGPT for learning English (Zou et al., 2025). Thus, the hypotheses were developed:

*H6*: FC positively influences PEOU.

*H7*: FC positively influences PU.

### 2.3 SI

SI is the process by which the real, imagined, or implied presence of other people alters an individual’s attitudes, beliefs, feelings, or behaviors (Mogaji et al., 2024). SI covers the many ways people affect one another, including through subtle pressure or direct commands (Lord and DeZoort, 2001). This process manifests in several common forms, including conformity, compliance, and obedience. Previous studies have shown positive influences of SI on PEOU and PU in explaining technology acceptance, such as artificial intelligence (Norzelan et al., 2024), telemedicine services (Vidal-Silva et al., 2024), and the metaverse (Wu and Yu, 2024). Thus, the hypotheses were developed:

*H8*: SI positively influences PEOU.

*H9*: SI positively influences PU.

### 2.4 PE

PE defines the extent to which the use of a specific technology is considered enjoyable in its own right, independent of any anticipated performance consequences (Wang and Lee, 2020). PE is a critical factor for technology acceptance. Previous studies have shown positive influences of PE on PEOU and PU in ChatGPT acceptance among students (Abdalla, 2024), AI-powered coding assistant acceptance among programmers (Kim et al., 2025), and online learning acceptance (Huang and Liu, 2024). Thus, the hypotheses were developed:

*H10*: PE positively influences PEOU.

*H11*: PE positively influences PU.

### 2.5 Contribution of mental health technology adoption and research gap

There were many studies on mental health technology adoption. For instance, researchers studied the adoption of mental health apps on mobile phones (Holtz et al., 2025). Also, the adoption of internet-based mental health technology has been studied (Philippi et al., 2021). Furthermore, the adoption of particular mental health wearable devices, such as smart headbands, wristbands, and watches, has been studied (Hunkin et al., 2020). However, no studies examined university students’ VR technology acceptance for improving mental health. Therefore, this study aimed to identify the determinants affecting university students’ acceptance of VR technology to improve mental health. Specifically, this study proposed a research model integrating the technology acceptance model (TAM), facilitating condition (FC), social influence (SI), and perceived enjoyment (PE). The findings were expected to enhance understanding of how FC, SI, and PE influence students’ acceptance of VR technology to improve mental health. This investigation also contributes to the literature by extending the applicability of the technology acceptance framework to the emerging context of VR-based mental health interventions among university populations, thereby addressing a gap at the intersection of digital mental health and technology adoption research. Moreover, this study would assist VR technology developers, educators, and policymakers in designing effective interventions to increase students’ acceptance of VR technology for improving mental health.

## 3 Methods

### 3.1 Measurements

To empirically test the study’s hypotheses, this study developed a self-administered questionnaire with three distinct sections. The first section captured demographic characteristics, such as gender, age, and VR experience. The second section assessed the core TAM constructs (PU, PEOU, ATU, and BITU), utilizing measurement items rigorously adapted from the literature to align with the specific context of VR-based mental health. The third section examined external influencing factors, including FC, SI, and PE, using scales derived from relevant prior studies. All measurement items were rated on a five-point Likert scale ranging from 1 (“strongly disagree”) to 5 (“strongly agree”), with pilot testing indicating an average completion time of approximately 10 min. Table 1 summarizes the measurement items.

**Table 1:** Measurement item summary.

Constructs	Items	Contents	References
Perceived ease of use (PEOU)	PEOU1	1. Using VR technology to improve mental health was easy for me.	Davis (1989) and Man et al. (2025b)
	PEOU2	2. Operating VR systems to improve mental health is easy for me.	
	PEOU3	3. Learning how to operate VR technology for improving mental health was easy to me.	
Perceived usefulness (PU)	PU1	1. Using VR technology would be useful for improving mental health.	Davis (1989) and Man et al. (2025b)
	PU2	2. Using VR technology would make mental health improvement more effective.	
	PU3	3. Using VR technology would improve my mental health.	
Attitude towards using (ATU)	AT1	1. Using VR technology for improving mental health is a good idea.	Davis (1989) and Man et al. (2025b)
	AT2	2. Using VR technology for improving mental health is a wise idea.	
	AT3	3. I feel positive about using VR technology for improving mental health.	
Facilitating condition (FC)	FC1	1. I believe a specific person will be available to help me in addressing the difficulties of using VR technology for improving mental health.	Al-Adwan et al. (2025)
	FC2	2. I believe proper guidance will be available when using VR technology for improving mental health.	
	FC3	3. I believe proper service is available if I face difficulty in using VR technology for improving mental health.	
Social influence (SI)	SI1	1. I will use VR technology for improving mental health if my family members and friends do so.	Man et al. (2025c)
	SI2	2. I will use VR technology for improving mental health if media/government encourages to use.	
	SI3	3. People who are important to me will support my use of VR technology for improving mental health.	
Perceived enjoyment (PE)	PE1	1. Using VR technology for improving mental health is fun.	Alalwan et al. (2018)
	PE2	2. Using VR technology for improving mental health is enjoyable.	
	PE3	3. Using VR technology for improving mental health is very entertaining.	
Behavioural intention to use (BITU)	BITU1	1. Assuming I can use VR technology to improve mental health, I intend to use it.	Davis (1989)
	BITU2	2. Given that I can use VR technology to improve mental health, I predict that I will use it.	
	BITU3	3. If I can use VR technology to improve mental health, I would like to use it as much as possible.	

The questionnaire items of this study were adapted from established English-language TAM-related scales and underwent a standardized cross-cultural adaptation process consistent with recommended guidelines (Wild et al., 2005). Specifically, the procedure included independent forward translation by two bilingual translators, reconciliation into a single Chinese version, independent back-translation by two additional bilingual translators blinded to the originals, and a committee review involving the research team and domain experts to resolve semantic, idiomatic, and conceptual discrepancies. Minor wording refinements were made to ensure cultural appropriateness for Chinese university students while preserving the constructs’ original meaning and psychometric integrity.

To address common method bias, a procedural approach was used following established guidelines in the literature (Kock, 2015). Specifically, items were worded clearly and neutrally to avoid ambiguity or leading effects. In addition, items from different constructs were intermixed to disrupt response patterns. Moreover, participants were assured of anonymity, confidentiality, and that there were no right or wrong answers, with voluntary participation emphasized.

### 3.2 Participants

This study adopted a non-probability sampling method for data collection from 327 university students across universities in Guangdong Province. Participants were approached during scheduled lecture breaks and in high-traffic campus areas (e.g., libraries and cafeterias). The non-probability convenience sampling method was employed to recruit university students, primarily due to practical constraints such as limited time, resources, and access to a comprehensive sampling frame of the target population. This method was deemed appropriate for the present exploratory study, which aimed to test relational mechanisms within an extended TAM framework in a relevant user group, rather than to estimate precise population parameters. Convenience sampling facilitated efficient data collection from accessible and willing participants, a common approach in similar cross-sectional technology acceptance research involving student samples (Aburbeian et al., 2022). However, the use of non-probability sampling may introduce potential selection bias because participants may differ systematically from non-participants. Consequently, external validity and generalizability of the findings are limited, and caution should be exercised when extending the results to the broader population of university students, particularly across diverse cultural, regional, or institutional contexts. Future studies are recommended to adopt probability-based sampling methods, such as stratified random sampling, or to employ multi-site designs to enhance representativeness and strengthen population-level inferences.

The inclusion criteria have now been clarified, including the following four parts. First, participants had to be currently enrolled university students (undergraduate or postgraduate) aged 18 or above. Second, they had to be able to understand and complete the questionnaire without cognitive limitations affecting response validity. Third, they had to have no prior experience with VR applications specifically designed for mental health. Last but not least, they had to report no conditions that could substantially interfere with their perception or interaction with VR content.

A brief explanation of the study purpose was provided to participants. Participants were given a standardized description explaining what VR mental health interventions are, including their definition, typical application scenarios, and illustrative examples, to ensure a common baseline understanding regardless of prior VR experience. Informed consent was obtained from all subjects involved in the study before administering the questionnaire. Furthermore, only participants without prior experience using VR mental health applications were included in this study to minimize potential confounding effects and ensure internal validity. The study was conducted in accordance with the Declaration of Helsinki, and approved by the Research Committee of the Guangzhou College of Commerce (approval number: 20250053).

Participants were recruited from multiple universities located across Guangdong province. Specifically, participants were recruited from universities representing diverse institutional types, including comprehensive universities (36%, 118 of 327), science and technology universities (39%, 127 of 327), normal universities (16%, 51 of 327), and private institutions (9%, 31 of 327). Regarding academic background, 68% of participants (223 of 327) were enrolled in STEM-related disciplines, whereas 32% (104 of 327) were from Arts, Humanities, or Social Science fields. Within the STEM group, majors included engineering (38%, 85 of 223), natural science (23%, 52 of 223), mathematics and statistics (21%, 47 of 223), and other disciplines (17%, 39 of 223). Within the ARTS group, majors included social sciences (33%, 34 of 104), business and management (31%, 32 of 104), humanities (28%, 29 of 104), and other disciplines (9%, 9 of 104). The final cohort consisted of 327 participants. Demographically, 44.3% were male, and 55.7% were female, with the majority aged between 18 and 22 years (mean = 20.1, SD = 1.3). The sample was generally tech-savvy, with 61.5% reporting prior VR experience.

### 3.3 Data analysis

Data analysis was conducted using AMOS 26 software, adhering to the two-step structural equation modeling (SEM) approach proposed by Anderson and Gerbing (1988). The first step employed confirmatory factor analysis (CFA) to validate the measurement model’s psychometric properties. In the second step, SEM was used to test the research hypotheses. Prior to structural path estimation, multicollinearity diagnostics were conducted using variance inflation factors (VIF). Model fit was evaluated against established benchmarks (Byrne, 2010), specifically requiring *χ*^2^/*df* < 5, CFI > 0.90, SRMR < 0.08, and RMSEA < 0.08. Furthermore, construct reliability and validity were rigorously established. Internal consistency was confirmed via Cronbach’s alpha (> 0.70) (Cronbach, 1951). Convergent validity was verified by an average variance extracted (AVE) exceeding 0.50 (Hamid et al., 2017). Discriminant validity was satisfied according to Fornell and Larcker (1981), which requires the square root of a construct’s AVE to surpass its correlations with all other constructs.

## 4 Results

### 4.1 Measurement model assessment

Table 2 presents the CFA results, confirming that the measurement model exhibits robust psychometric properties suitable for SEM. The model fit indices (*χ*^2^/*df* = 2.477, CFI = 0.979, TLI = 0.974, and RMSEA = 0.067) satisfied all recommended benchmarks, indicating a strong fit to the data. Convergent validity and internal consistency were substantiated, as all factor loadings (FL) and composite reliability (CR) scores exceeded the 0.70 threshold, AVE values surpassed 0.50, and Cronbach’s alpha coefficients ranged from 0.79 to 0.98. Furthermore, discriminant validity was established (Table 3), as the square root of the AVE for each construct exceeded its bivariate correlations with all other constructs. The results showed that all VIF values fall within the commonly accepted range of 1–10 reported in prior methodological research, indicating no multicollinearity (O’brien, 2007). Although the PU − ATU correlation is strong, it does not necessarily indicate construct redundancy. Prior technology acceptance literature has established that PU and ATU are theoretically distinct constructs despite being closely related (Venkatesh and Thong, 2016). The observed relationship may therefore reflect a substantively meaningful association consistent with earlier findings showing that individuals with higher PU tend to exhibit more favorable attitudes toward emerging technologies (Holden and Rada, 2011).

**Table 2:** Measurement model assessment results.

Constructs	Items	Mean	SD	FL	AVE	CR	Cronbach’s alpha
Perceived ease of use (PEOU)	PEOU1	3.664	0.986	0.811	0.829	0.935	0.931
	PEOU2	3.581	0.987	0.960			
	PEOU3	3.679	0.993	0.952			
Perceived usefulness (PU)	PU1	3.645	0.970	0.978	0.947	0.982	0.982
	PU2	3.618	1.002	0.976			
	PU3	3.578	1.009	0.967			
Attitude towards using (ATU)	AT1	3.749	0.974	0.983	0.946	0.981	0.981
	AT2	3.691	0.990	0.984			
	AT3	3.780	0.979	0.949			
Facilitating condition (FC)	FC1	3.131	1.174	0.874	0.832	0.937	0.936
	FC2	3.272	1.128	0.949			
	FC3	3.281	1.127	0.913			
Social influence (SI)	SI1	3.306	0.916	0.980	0.842	0.941	0.937
	SI2	3.269	0.907	0.959			
	SI3	3.483	0.892	0.803			
Perceived enjoyment (PE)	PE1	3.783	1.018	0.970	0.933	0.977	0.977
	PE2	3.813	1.047	0.949			
	PE3	3.719	1.021	0.979			
Behavioural intention to use (BITU)	BITU1	3.596	0.964	0.963	0.937	0.978	0.978
	BITU2	3.621	0.948	0.986			
	BITU3	3.590	0.961	0.955			

**Table 3:** Correlations among constructs.

	PEOU	PU	ATU	FC	SI	PE	BITU	VIF
PEOU	0.910							4.287
PU	0.856***	0.973						7.945
ATU	0.846***	0.953***	0.972					5.873
FC	0.639***	0.693***	0.646***	0.912				2.142
SI	0.630***	0.705***	0.685***	0.650***	0.918			8.559
PE	0.826***	0.897***	0.915***	0.587***	0.662***	0.966		3.416
BITU	0.738***	0.840***	0.855***	0.600***	0.764***	0.797***	0.968	

SAVEs of the constructs are on the diagonal. ***p < 0.001.

### 4.2 Structural model assessment

**Figure 1:**
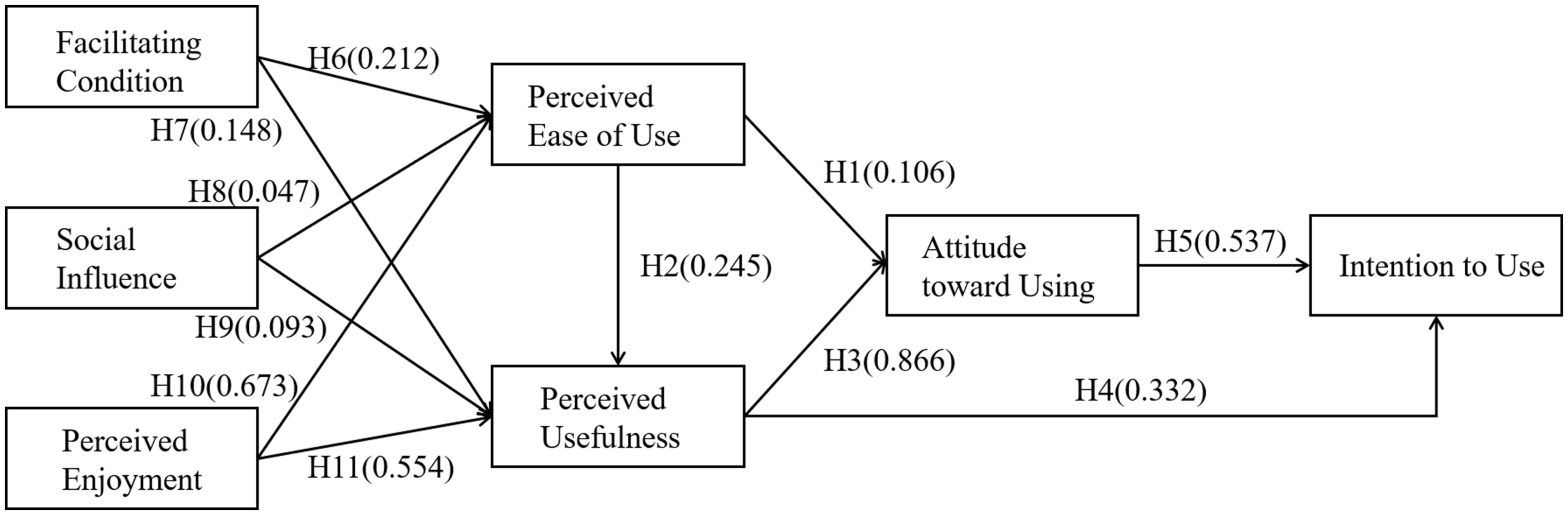
Structural model with path coefficients.

The structural model was assessed using the same fit criteria applied to the measurement model, with all indices (*χ*^2^/*df* = 2.995, CFI = 0.970, TLI = 0.964, and RMSEA = 0.078) meeting recommended benchmarks (Kline, 2023). This result confirmed that the model adequately represents the hypothesized relationships. As summarized in Figure 1 and Table 4, which details the path coefficients and testing outcomes, 10 of the 11 formulated hypotheses received empirical support. Specifically, PEOU and PU positively influenced ATU, supporting H1 and H3. PEOU positively influenced PU, supporting H2. PU and ATU positively influenced BITU, supporting H4 and H5. FC and PE positively influenced PEOU and PU, supporting H6, H7, H10, and H11. SI positively influenced PU but not PEOU, supporting H9 and rejecting H8. The structural model can explain 72.3, 88.0, 91.9, and 74.0% of the variance for PEOU, PU, ATU, and BITU, respectively.

**Table 4:** Hypothesis testing results.

Hypotheses	Standardised path coefficients	*p*-values
H1: PEOU → ATU (+)	0.106	0.007
H2: PEOU → PU (+)	0.245	< 0.001
H3: PU → ATU (+)	0.866	< 0.001
H4: PU → BITU (+)	0.332	0.005
H5: ATU → BITU (+)	0.537	< 0.001
H6: FC → PEOU (+)	0.212	< 0.001
H7: FC → PU (+)	0.148	< 0.001
H8: SI → PEOU (+)	0.047	0.343
H9: SI → PU (+)	0.093	0.004
H10: PE → PEOU (+)	0.673	< 0.001
H11: PE → PU (+)	0.554	< 0.001

## 5 Discussion

This study developed an extended TAM integrating FC, SI, and PE to address existing limitations in the literature regarding VR-based mental health interventions. The extended TAM posits that VR technology acceptance among university students for improving mental health is primarily driven by PEOU and PU, which are positively influenced by FC, SI, and PE. By successfully validating the extended TAM, this study deepened the understanding of adoption determinants and made a large contribution to the literature on VR-based mental health interventions. This study ultimately offered practical recommendations to increase the uptake of VR technology among university students for mental health interventions.

### 5.1 Theoretical implications

This study represents the pioneering integration of FC, SI, and PE into the TAM framework to explain university students’ VR technology acceptance for mental health improvement. By confirming that PEOU drives PU and that both constructs, mediated by ATU, significantly influence BITU, this study validated the theoretical robustness of the TAM in this specific domain. These results aligned with recent findings by Man et al. (2025a,b) regarding VR technology in secondary education, thereby extending the application of the TAM from academic learning to mental health interventions and solidifying its role in predicting university student’ engagement with emerging technologies.

FC was found to positively influence PEOU and PU in the context of VR technology acceptance among university students for improving mental health. These findings were consistent with Man et al. (2025c), who found that PEOU and PU were positively influenced by FC for the public to adopt highly automated vehicles. The findings indicated that FC, such as accessible hardware, reliable technical support, and a private and secure environment, is a crucial antecedent for university students adopting VR technology for improving mental health. When these structural supports are robust, university students view VR technology as less complex and easy to navigate because technical friction is removed from the therapeutic process (Teng et al., 2022). Furthermore, this seamless user experience allows university students to focus on the actual psychological benefits rather than interface struggles, leading them to perceive the VR-based interventions as highly effective and valuable for their mental health.

This study showed that SI positively affected PU but not PEOU in the context of VR technology acceptance among university students for improving mental health. These findings partly aligned with those of Zhang et al. (2020), who found that PU and PEOU were positively influenced by SI for the public to adopt automated vehicles. The findings suggested that SI stemming from peer endorsement serves as a form of social validation that significantly enhances university students’ PU of VR technology for improving mental health. University students are likely to believe in the therapeutic value of VR-based interventions if they are socially recommended by peers (Glaus et al., 2023). However, this study diverged from Zhang et al. (2020) by finding no link between SI and PEOU, likely because the operation of VR hardware is a hands-on and individual technical skill. Different from automated vehicles, where social acceptance can reduce anxiety and imply effortless use (Man et al., 2025c), peer endorsement of VR technology does not practically reduce the learning curve or technical complexity required to operate VR headsets.

This study discovered that PE positively influenced PEOU and PU in the context of VR technology acceptance among university students for improving mental health. These findings were consistent with those in ChatGPT acceptance among students (Abdalla, 2024), AI-powered coding assistant acceptance among programmers (Kim et al., 2025), and online learning acceptance (Huang and Liu, 2024). The findings underscored the dual impact of PE as a vital intrinsic motivator that lowers technical barriers while enhancing university students’ perceived therapeutic value of VR-based interventions for improving mental health. When university students find the VR environment inherently pleasurable or immersive, the cognitive load associated with navigating the interface is psychologically mitigated, leading to high PEOU (Wenk et al., 2023). Simultaneously, this enjoyment amplifies university students’ PU of VR-based interventions for improving mental health because high engagement and positive affect are key indicators of efficacy of VR-based interventions in the context of mental health (Pira et al., 2023). If VR-based interventions are enjoyable, university students view VR-based interventions as valid and effective for emotional regulation or stress relief rather than just a novelty.

### 5.2 Practical implications

Three practical suggestions were discussed based on the findings of this study to increase university students’ use of VR technology to improve mental health. First, this study indicated the positive effects of FC on university students’ PEOU and PU. Universities should implement dedicated and private zones equipped with high-fidelity and pre-configured VR headsets that require zero setup from users to eliminate technical friction and maximize PEOU (Bell et al., 2024). Furthermore, providing on-demand technical support and intuitive and quick-start guides is essential to ensure that university students can focus entirely on the therapeutic content rather than troubleshooting hardware. Second, based on the finding that SI drives PU, universities should use social channels to validate the value of VR technology by establishing a mental health ambassador program (Fung et al., 2022). In the program, trusted peers and faculty share testimonials to socially validate the VR technology efficacy in improving mental health. Third, to leverage the impacts of PE on PEOU and PU, universities and developers should prioritize therapeutic gamification over clinical interfaces when selecting or designing VR-based mental health applications (Cheng et al., 2025; Kim and Choi, 2025). The applications should integrate immersive narrative arcs, interactive calming environments (such as dynamic nature landscapes), and subtle reward systems to maintain consistency because these elements transform the user experience from a medical task into an engaging activity for university students. Last, to foster a positive attitude of university students toward VR technology for improving mental health, VR-based intervention developers must adopt a dual-strategy that prioritizes intuitive design alongside clear value communication (Lan et al., 2025). Practically, VR-based intervention developers should invest in rigorous user experience testing to simplify the interface and minimize the learning curve for university students (Sun and Jiang, 2025). Simultaneously, developers should explicitly market the tangible outcomes of VR-based interventions, such as specific stress-reduction metrics or testimonial success stories, to demonstrate that the interventions are not just a novelty but a highly effective solution.

### 5.3 Limitations

Although this study contributed to the theory and practice of VR-based mental health interventions, several limitations should be pointed out. First, the reliance on a cross-sectional design offers only a static snapshot, potentially failing to capture the dynamic nature of VR technology adoption among university students. Consequently, future longitudinal research is recommended to examine how these interrelationships evolve. Second, although personality traits are recognized as critical determinants of attitude (Calluso and Devetag, 2025), they were outside the scope of this specific investigation. Future research should therefore integrate these variables to provide a comprehensive explanation of university students’ acceptance of VR technology for improving mental health. Third, all participants had no prior experience using VR mental health applications, and approximately 40% had no prior exposure to VR at all. Their responses, therefore, relied on the provided descriptions rather than direct interaction, which may have influenced perception-based measures and the results. Future studies should incorporate hands-on exposure or experimental designs to enhance the ecological validity of the findings. As the sample was drawn from university students within a single regional context, caution should be exercised when generalizing the findings to other populations, educational systems, or cultural settings. Further research using more diverse samples is warranted to strengthen external validity.

## Data Availability

The raw data supporting the conclusions of this article will be made available by the authors, without undue reservation.

## References

[ref1] Abdalla R. A. (2024). Examining awareness, social influence, and perceived enjoyment in the TAM framework as determinants of ChatGPT. personalization as a moderator. J. Open Innov. Technol. Mark. Complex. 10:100327. doi: 10.1016/j.joitmc.2024.100327

[ref2] Abulfaraj G. G. Upsher R. Zavos H. M. Dommett E. J. (2024). The impact of resilience interventions on university students’ mental health and well-being: a systematic review. Educ. Sci. 14:510. doi: 10.3390/educsci14050510

[ref3] Aburbeian A. M. Owda A. Y. Owda M. (2022). A technology acceptance model survey of the metaverse prospects. AI 3, 285–302. doi: 10.3390/ai3020018

[ref4] Al-Adwan A. S. Meet R. K. Anand S. Shukla G. P. Alsharif R. Dabbaghia M. (2025). Understanding continuous use intention of technology among higher education teachers in emerging economy: evidence from integrated TAM, TPACK, and UTAUT model. Stud. High. Educ. 50, 505–524.

[ref5] Alalwan A. A. Baabdullah A. M. Rana N. P. Tamilmani K. Dwivedi Y. K. (2018). Examining adoption of mobile internet in Saudi Arabia: extending TAM with perceived enjoyment, innovativeness and trust. Technol. Soc. 55, 100–110. doi: 10.1016/j.techsoc.2018.06.007

[ref6] Anderson J. C. Gerbing D. W. (1988). Structural equation modeling in practice: a review and recommended two-step approach. Psychol. Bull. 103:411. doi: 10.1037/0033-2909.103.3.411

[ref7] Auerbach R. P. Mortier P. Bruffaerts R. Alonso J. Benjet C. Cuijpers P. . (2018). WHO world mental health surveys international college student project: prevalence and distribution of mental disorders. J. Abnorm. Psychol. 127:623. doi: 10.1037/abn0000362, 30211576 PMC6193834

[ref8] Bell I. H. Pot-Kolder R. Rizzo A. Rus-Calafell M. Cardi V. Cella M. . (2024). Advances in the use of virtual reality to treat mental health conditions. Nat. Rev. Psychol. 3, 552–567. doi: 10.1038/s44159-024-00334-9

[ref9] Byrne B. M. (2010). Structural Equation Modeling with AMOS: Basic Concepts, Applications, and Programming. 2nd Edn. New York: Routledge/Taylor & Francis Group.

[ref10] Calluso C. Devetag M. G. (2025). The impact of technology acceptance and personality traits on the willingness to use AI-assisted hiring practices. Int. J. Organ. Anal. 33, 1368–1385. doi: 10.1108/IJOA-06-2024-4562

[ref11] Cerolini S. Zagaria A. Franchini C. Maniaci V. G. Fortunato A. Petrocchi C. . (2023). Psychological counseling among university students worldwide: a systematic review. Eur. J. Investig. Health Psychol. Educ. 13, 1831–1849. doi: 10.3390/ejihpe13090133, 37754472 PMC10528000

[ref12] Cheng J. Lu C. Xiao Q. (2025). Effects of gamification on EFL learning: a quasi-experimental study of reading proficiency and language enjoyment among Chinese undergraduates. Front. Psychol. 16:1448916. doi: 10.3389/fpsyg.2025.1448916, 40171076 PMC11958712

[ref13] Cronbach L. J. (1951). Coefficient alpha and the internal structure of tests. Psychometrika 16, 297–334. doi: 10.1007/bf02310555

[ref14] Davis F. D. (1989). Perceived usefulness, perceived ease of use, and user acceptance of information technology. MIS Q. 13, 319–340. doi: 10.2307/249008

[ref15] Fornell C. Larcker D. F. (1981). Evaluating structural equation models with unobservable variables and measurement error. J. Mark. Res. 18, 39–50. doi: 10.1177/002224378101800104

[ref16] Fung K. P.-L. Liu J. J. Sin R. Bender A. Shakya Y. Butt N. . (2022). Exploring mental illness stigma among Asian men mobilized to become community mental health ambassadors in Toronto Canada. Ethn. Health 27, 100–118. doi: 10.1080/13557858.2019.1640350 31339347

[ref17] Glaus C. E. Kloeti A. Vokinger K. N. (2023). Defining ‘therapeutic value’of medicines: a scoping review. BMJ Open 13:e078134. doi: 10.1136/bmjopen-2023-078134, 38110384 PMC10748878

[ref18] Hamid M. R. A. Sami W. Sidek M. H. M. (2017). Discriminant validity assessment: use of Fornell & Larcker criterion versus HTMT criterion. J. Phys. Conf. Ser. 890:012163. doi: 10.1088/1742-6596/890/1/012163

[ref19] Holden H. Rada R. (2011). Understanding the influence of perceived usability and technology self-efficacy on teachers’ technology acceptance. J. Res. Technol. Educ. 43, 343–367. doi: 10.1080/15391523.2011.10782576

[ref20] Holtz B. E. Kanthawala S. Martin K. Nelson V. Parrott S. (2025). Young adults’ adoption and use of mental health apps: efficient, effective, but no replacement for in-person care. J. Am. Coll. Heal. 73, 602–610. doi: 10.1080/07448481.2023.2227727, 37399569

[ref21] Huang F. Liu S. (2024). If i enjoy, i continue: the mediating effects of perceived usefulness and perceived enjoyment in continuance of asynchronous online English learning. Educ. Sci. 14:880. doi: 10.3390/educsci14080880

[ref22] Hunkin H. King D. L. Zajac I. T. (2020). Perceived acceptability of wearable devices for the treatment of mental health problems. J. Clin. Psychol. 76, 987–1003. doi: 10.1002/jclp.22934, 32022908

[ref23] Kim Y. W. Cha M. C. Yoon S. H. Lee S. C. (2025). Not merely useful but also amusing: impact of perceived usefulness and perceived enjoyment on the adoption of AI-powered coding assistant. Int. J. Hum. Comput. Interact. 41, 6210–6222. doi: 10.1080/10447318.2024.2375701

[ref24] Kim H. Choi Y. (2025). Developing interactive VR-based digital therapeutics for acceptance and commitment therapy (ACT): a structured framework for the digital transformation integrating gamification and multimodal arts. Front. Psych. 16:1554394. doi: 10.3389/fpsyt.2025.1554394, 40534907 PMC12174457

[ref001] Kline R. B. (2023). Principles and practice of structural equation modeling. Guilford publications.

[ref25] Kock N. (2015). Common method bias in PLS-SEM: a full collinearity assessment approach. Int. J. e-Collab. 11, 1–10. doi: 10.4018/ijec.2015100101

[ref26] Kuleli D. Tyson P. Davies N. H. Zeng B. (2025). Examining the comparative effectiveness of virtual reality and in-vivo exposure therapy on social anxiety and specific phobia: a systematic review & meta-analysis. J. Behav. Cogn. Ther. 35:100524. doi: 10.1016/j.jbct.2025.100524

[ref27] Lan Y. Liu S. Chen H. Xia L. (2025). Mindfulness and AI adoption: extending the technology acceptance model for Chinese media students. Front. Psychol. 16:1637502. doi: 10.3389/fpsyg.2025.1637502, 40969467 PMC12442562

[ref28] Li X. Hu Y. Yang X. Bi X. Zhang J. Tao P. (2025). The effectiveness of virtual reality training on social skills in education: a meta-analysis. Educ. Inf. Technol. 30, 89–105. doi: 10.1007/s10639-024-12941-3

[ref29] Liu C. Wang Y. Evans M. Correia A.-P. (2024). Critical antecedents of mobile learning acceptance and moderation effects: a meta-analysis on technology acceptance model. Educ. Inf. Technol. 29, 20351–20382. doi: 10.1007/s10639-024-12645-8

[ref30] Lord A. T. DeZoort F. T. (2001). The impact of commitment and moral reasoning on auditors' responses to social influence pressure. Account. Organ. Soc. 26, 215–235. doi: 10.1016/s0361-3682(00)00022-2

[ref31] Man S. S. Ding M. Li X. Chan A. H. S. Zhang T. (2025c). Acceptance of highly automated vehicles: the role of facilitating condition, technology anxiety, social influence and trust. Int. J. Hum. Comput. Interact. 41, 3684–3695. doi: 10.1080/10447318.2024.2316389

[ref32] Man S. S. Fang Y. Chan A. H. S. Han J. (2025a). Exploring the acceptance of learning English through VR technology amongst secondary school students: intrinsic and extrinsic motivation as key determinants. J. Educ. Comput. Res. 64:07356331251390717. doi: 10.1177/07356331251390717

[ref33] Man S. S. Fang Y. Chan A. H. S. Han J. (2025b). VR technology acceptance for English learning amongst secondary school students: role of classroom climate and language learning anxiety. Educ. Inf. Technol. 30, 4131–4155. doi: 10.1007/s10639-024-12969-5

[ref34] Man S. S. Guo Y. Chan A. H. S. Zhuang H. (2022). Acceptance of online mapping technology among older adults: technology acceptance model with facilitating condition, compatibility, and self-satisfaction. ISPRS Int. J. Geo Inf. 11:558. doi: 10.3390/ijgi11110558

[ref35] Man S. S. Li X. Lin X. J. Lee Y.-C. Chan A. H. S. (2025d). Assessing the effectiveness of virtual reality interventions on anxiety, stress, and negative emotions in college students: a meta-analysis of randomized controlled trials. Int. J. Hum. Comput. Interact. 41, 10495–10511. doi: 10.1080/10447318.2024.2434957

[ref36] Man S. S. Wen H. So C. L. (2024). Are virtual reality applications effective for construction safety training and education? A systematic review and meta-analysis. J. Saf. Res. 88, 230–243. doi: 10.1016/j.jsr.2023.11.011, 38485365

[ref37] Mogaji E. Viglia G. Srivastava P. Dwivedi Y. K. (2024). Is it the end of the technology acceptance model in the era of generative artificial intelligence? Int. J. Contemp. Hosp. Manag. 36, 3324–3339. doi: 10.1108/ijchm-08-2023-1271

[ref38] Mortier P. Cuijpers P. Kiekens G. Auerbach R. P. Demyttenaere K. Green J. G. . (2018). The prevalence of suicidal thoughts and behaviours among college students: a meta-analysis. Psychol. Med. 48, 554–565. doi: 10.1017/S0033291717002215 28805169

[ref39] Mu L. Du B. Hou X. (2022). A study on the improvement of college students’ psychological pressure and anxiety by using English psychological script activities. Front. Psychol. 13:878479. doi: 10.3389/fpsyg.2022.878479, 35572300 PMC9094481

[ref40] Norzelan N. A. Mohamed I. S. Mohamad M. (2024). Technology acceptance of artificial intelligence (AI) among heads of finance and accounting units in the shared service industry. Technol. Forecast. Soc. Change 198:123022. doi: 10.1016/j.techfore.2023.123022

[ref41] O’brien R. M. (2007). A caution regarding rules of thumb for variance inflation factors. Qual. Quant. 41, 673–690. doi: 10.1007/s11135-006-9018-6

[ref42] Olasz O. Erdős S. Horváth K. (2024). The effects of virtual reality-based mindfulness exercises on the perception of time, psychological and physiological states of young people: a randomized crossover trial. Mindfulness 15, 2347–2354. doi: 10.1007/s12671-024-02438-y

[ref43] Ozamiz-Etxebarria N. Idoiaga Mondragon N. Tsybuliak N. (2025). Strengthening mental health among university students. Front. Psychol. 16:1689173. doi: 10.3389/fpsyg.2025.1689173, 41280192 PMC12630115

[ref44] Philippi P. Baumeister H. Apolinário-Hagen J. Ebert D. D. Hennemann S. Kott L. . (2021). Acceptance towards digital health interventions–model validation and further development of the unified theory of acceptance and use of technology. Internet Interv. 26:100459. doi: 10.1016/j.invent.2021.100459, 34603973 PMC8463857

[ref45] Pira G. L. Aquilini B. Davoli A. Grandi S. Ruini C. (2023). The use of virtual reality interventions to promote positive mental health: systematic literature review. JMIR Ment. Health 10:e44998. doi: 10.2196/44998 37410520 PMC10360019

[ref46] Sharma B. K. Kumar V. R. Bhatt V. K. (2024). Factors influencing E-learning technology among youth in India: an extended TAM model. Manag. Labour Stud. 49, 504–526. doi: 10.1177/0258042X231208588

[ref47] Shi X. Pan J. Yuan D. Li M. Pan Y. (2025). Advanced data analysis and prediction model for student mental health risk assessment. Front. Psychol. 16:1682083. doi: 10.3389/fpsyg.2025.1682083, 41307019 PMC12644000

[ref48] Sun N. Jiang Y. (2025). Eye movements and user emotional experience: a study in interface design. Front. Psychol. 16:1455177. doi: 10.3389/fpsyg.2025.1455177, 40177033 PMC11961869

[ref49] Teng Z. Cai Y. Gao Y. Zhang X. Li X. (2022). Factors affecting learners’ adoption of an educational metaverse platform: an empirical study based on an extended UTAUT model. Mob. Inf. Syst. 2022:5479215. doi: 10.1155/2022/5479215

[ref50] Venkatesh V. Thong J. (2016). Unified theory of acceptance and use of technology: a synthesis and the road ahead. J. Assoc. Inf. Syst. 17, 328–376. doi: 10.17705/1jais.00428

[ref51] Vidal-Silva C. Sánchez-Ortiz A. Serrano-Malebrán J. Arriagada V. Flores M. Godoy M. . (2024). Social influence, performance expectancy, and price value as determinants of telemedicine services acceptance in Chile. Heliyon 10:e27067. doi: 10.1016/j.heliyon.2024.e27067, 38562504 PMC10982984

[ref52] Wandira R. Fauzi A. Nurahim F. (2024). Analysis of factors influencing behavioral intention to use cloud-based academic information system using extended technology acceptance model (TAM) and expectation-confirmation model (ECM). J. Inf. Syst. Eng. Bus. Intell. 10, 179–190. doi: 10.20473/jisebi.10.2.179-190

[ref53] Wang H. Lee K. (2020). Getting in the flow together: the role of social presence, perceived enjoyment and concentration on sustainable use intention of mobile social network game. Sustainability 12:6853. doi: 10.3390/su12176853

[ref54] Wenk N. Penalver-Andres J. Buetler K. A. Nef T. Müri R. M. Marchal-Crespo L. (2023). Effect of immersive visualization technologies on cognitive load, motivation, usability, and embodiment. Virtual Reality 27, 307–331. doi: 10.1007/s10055-021-00565-8, 36915633 PMC9998603

[ref55] Wild D. Grove A. Martin M. Eremenco S. McElroy S. Verjee-Lorenz A. . (2005). Principles of good practice for the translation and cultural adaptation process for patient-reported outcomes (PRO) measures: report of the ISPOR task force for translation and cultural adaptation. Value Health 8, 94–104. doi: 10.1111/j.1524-4733.2005.04054.x, 15804318

[ref56] Wu R. Yu Z. (2024). Investigating users’ acceptance of the metaverse with an extended technology acceptance model. Int. J. Hum. Comput. Interact. 40, 5810–5826. doi: 10.1080/10447318.2023.2241295

[ref57] Zhang T. Tao D. Qu X. Zhang X. Zeng J. Zhu H. . (2020). Automated vehicle acceptance in China: social influence and initial trust are key determinants. Transp. Res. Part C Emerg. Technol. 112, 220–233. doi: 10.1016/j.trc.2020.01.027

[ref58] Zou B. Wang C. Yan Y. Du X. Ji Y. (2025). Exploring English as a foreign language learners’ adoption and utilisation of ChatGPT for speaking practice through an extended technology acceptance model. Int. J. Appl. Linguist. 35, 689–704. doi: 10.1111/ijal.12658

